# Electrochemical corrosion assessment of RaCe and Mtwo rotary nickle-titanium instruments after clinical use and sterilization

**DOI:** 10.4317/medoral.17413

**Published:** 2011-12-06

**Authors:** Shahriar Shahi, Hadi Mokhtari, Saeed Rahimi, Vahab Shiezadeh, Habib Ashasi, Majid Abdolrahimi, Mohammad Foroughreyhani

**Affiliations:** 1 DDS, MSc, Associate Professor, Department of Endodontics,Faculty of Dentistry, Dental and Periodontal Research Center, Tabriz University of Medical Sciences, Tabriz, Iran; 2 DDS, MSc, Assistant Professor, Department of Endodontics, Faculty of Dentistry, Dental and Periodontal Research Center, Tabriz University of Medical Sciences, Tabriz, Iran; 3 PHD, Department of Electrochemistry, Faculty of Chemistry, Tabriz University, Tabriz, Iran

## Abstract

Aim: The aim of the present study was to electrochemically evaluate corrosion resistance of RaCe and Mtwo files after repeated sterilization and preparation procedures. Study Design: A total of 450 rotary files were used. In the working groups, 72 files from each file type were distributed into 4 groups. RaCe and Mtwo files were used to prepare one root canal of the mesial root of extracted human mandibular first molars. The procedure was repeated to prepare 2 to 8 canals. The following irrigation solutions were used: group 1, RaCe files with 2.5% NaOCl; group 2, RaCe files with normal saline; group 3, Mtwo files with 2.5% NaOCl; and group 4, Mtwo files with normal saline in the manner described. In autoclave groups, 72 files from each file type were evenly distributed into 2 groups. Files were used for a cycle of sterilization without the use of files for root canal preparation. Nine new unused files from each file type were used as controls. Then the instruments were sent for corrosion assessment. Mann-Whitney U and Wilcoxon tests were used for independent and dependent groups, respectively. Results: Statistical analysis indicated that there were significant differences in corrosion resistance of files associated with working and autoclave groups between RaCe and Mtwo file types (p<0.001). Conclusions: Corrosion resistance of #25, #30, and #35 Mtwo files is significantly higher than that in RaCe files with similar sizes.

** Key words:**Corrosion, NiTi instruments, autoclave, RaCe, Mtwo.

## Introduction

The potential fracture risk of nickel-titanium (NiTi) endodontic rotary instruments during root canal shaping is of great concern (1). The chemical mechanisms that occur, either prior to or during instrumentation may cause corrosion of the instruments, leading to early failure (2). Sodium hypochlorite is the most commonly used irrigant in endodontic treatment; it is delivered to the apical part of canal to carry out its bactericidal action and to dissolve organic substances (3,4). NaOCl can lead to micro-pitting if used as a disinfectant when cleaning NiTi file (5). Stokes et al. (6) described pitting corrosion of the file surface after 1 hour of immersion in 5.25% NaOCl and speculated that manufacturing factors might adversely affect the corrosion behavior of root canal instruments. NaOCl contains active ClO- ions, which increase corrosion potential (7). On the other hand, NaOCl treatment does not alter the cutting efficacy of NiTi instruments, does not lead to a significant decrease in flexural or torsional strength of NiTi instruments after immersion in NaOCl, and does not adversely affect cyclic fatigue of NiTi instruments after multiple clinical uses (8,9). Corrosion mechanisms might be activated during chemo-mechanical preparation, cleaning procedures, chemical disinfection, or sterilization (2,10). The corrosion resistance of NiTi alloy can be enhanced by lowering the pH of NaOCl solution (11). Several investigators have demonstrated a correlation between changes in surface chemical composition of NiTi files and several sterilization treatments and working loads due to several root canal preparation procedures with an instrument (12-14). When assessing corrosion of rotary Ni-Ti files, it must be taken into account that corrosion resistance of such instruments depends on a broad range of conditions. Some studies have shown the effect of these conditions on both new and used NiTi instruments (8); however, it is necessary to evaluate the effect of possible factors on corrosive behavior of files in situations as close to clinical situations as possible. There are no reports in the literature about corrosion failure of Mtwo and RaCe files. Therefore, the aim of this study was to examine the effect of several cycles of root canal preparation and sterilization procedures on nickel-titanium rotary files from two different manufacturers and to determine whether corrosion resistance of these files is influenced by the number of clinical uses and sterilization procedures.

## Material and Methods

In the present study a total of 450 NiTi rotary endodontic files were used; half of the files were RaCe rotary files (FKG, La-chaux-de-fonds, Switzerland) and half were Mtwo rotary files (VDW, Munchen, Germany), which were dispensed from their original packaging and immediately used for the purpose of the study. The files were randomly divided into three groups working, autoclave and control as follows:

1. Working group: A total of 288 files were used in this group, half of which (n=144) were RaCe and half were Mtwo files.

In the RaCe group the files were divided into 16 subgroups of A1–A8 and B1–B8. In each subgroup a new package of files consisting of 9 files was used as follows:

Three #25 files with a taper of 0.06; three #30 files with a taper of 0.06; and three #35 files with a taper of 0.04.

In the subgroups A1–A8 and B1–B8, 2.5% NaOCl at 50°C and normal saline (0.9% NaCl) were used as canal irrigants, respectively.

In the subgroups A1– A8 and B1–B8, each file was used once (A1 and B1) to eight times (A8 and B8) to prepare one mesiobuccal canal of a mandibular first molar. Each file was disinfected with 2.5% NaOCl at 50°C for 5 minutes and then autoclaved at 121°C, 15 PSI for 15 minutes after each use.

In the Mtwo group the same procedures were repeated for subgroups C1– C8 and D1–D8. Mtwo packages consisted of 9 files as follows:

Three #25 files with a taper of 0.06; three #30 files with a taper of 0.05; and three #35 files with a taper of 0.04 ( Table 1).

**Table 1:**
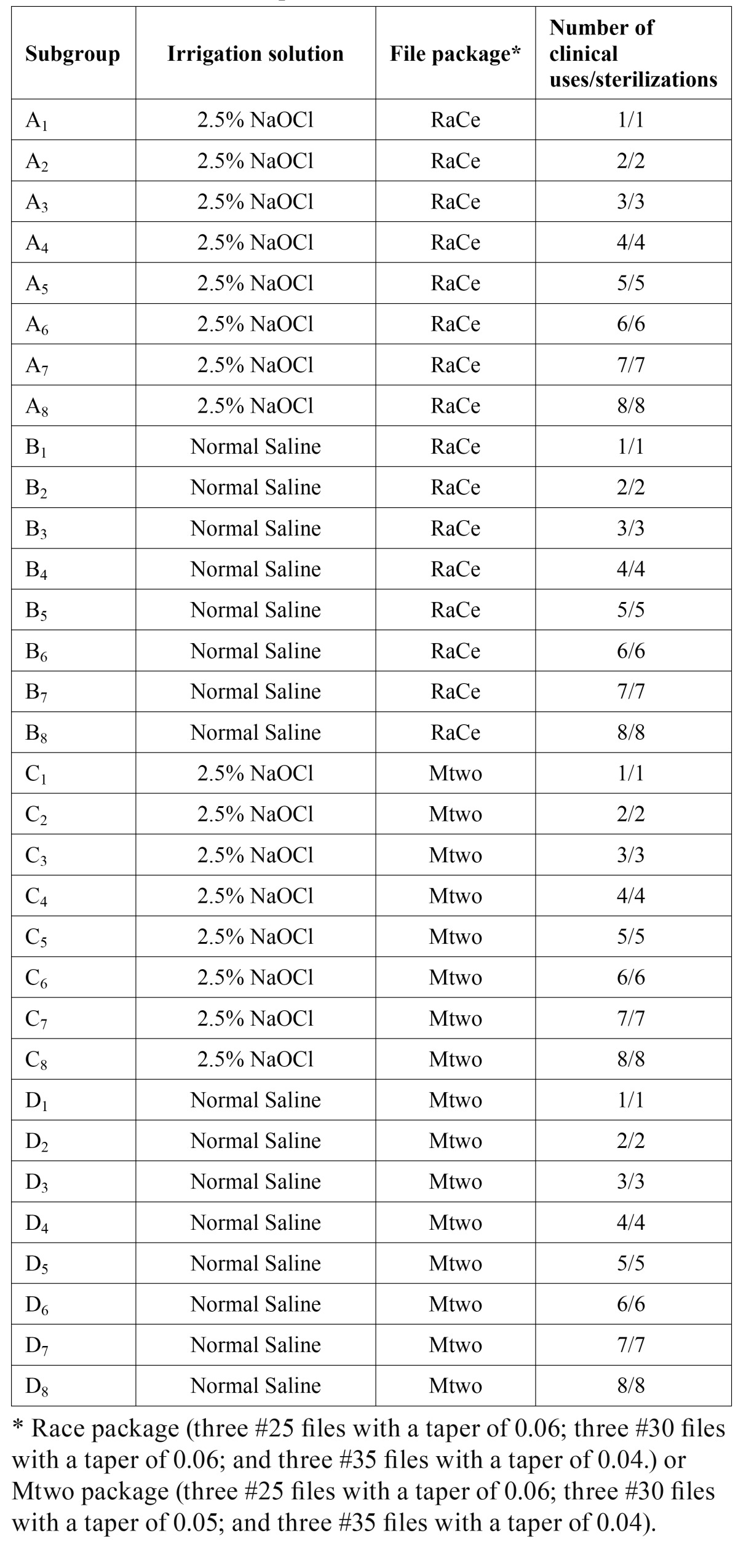
Working subgroups, irrigation solutions and number of clinical and sterilization procedures used (n=288).

Mesiobuccal canals of mandibular first molars extracted for periodontal reasons were used in the present study. The maximum canal curvature was 20°, as determined by the Schneider method. All the roots were the same length and the working length was determined by K-files #15 (Dentsply/Maillefer, Ballagigues, Switzerland) after accessing the cavity preparation with a tapered fissure bur (D&Z, Wiesbaden, Germany). The working length was recorded 1 mm less when the file tip just emerged through the apical foramen.

2. Autoclave group: A total of 144 NiTi rotary files were used in this group, half of which (n=72) were RaCe (subgroups E1–E8) and half were Mtwo (subgroups F1–F8). The files in the subgroups E1–E8 and F1–F8 were disinfected and autoclaved without canal preparation before the corrosion analysis as follows:

E1 and F1 (once), E2 and F2 (twice), E3 and F3 (three times), etc, and E8 and F8 (eight times).

The same RaCe and Mtwo file packages of 9 files were used similar to the working groups ( Table 2).

**Table 2:**
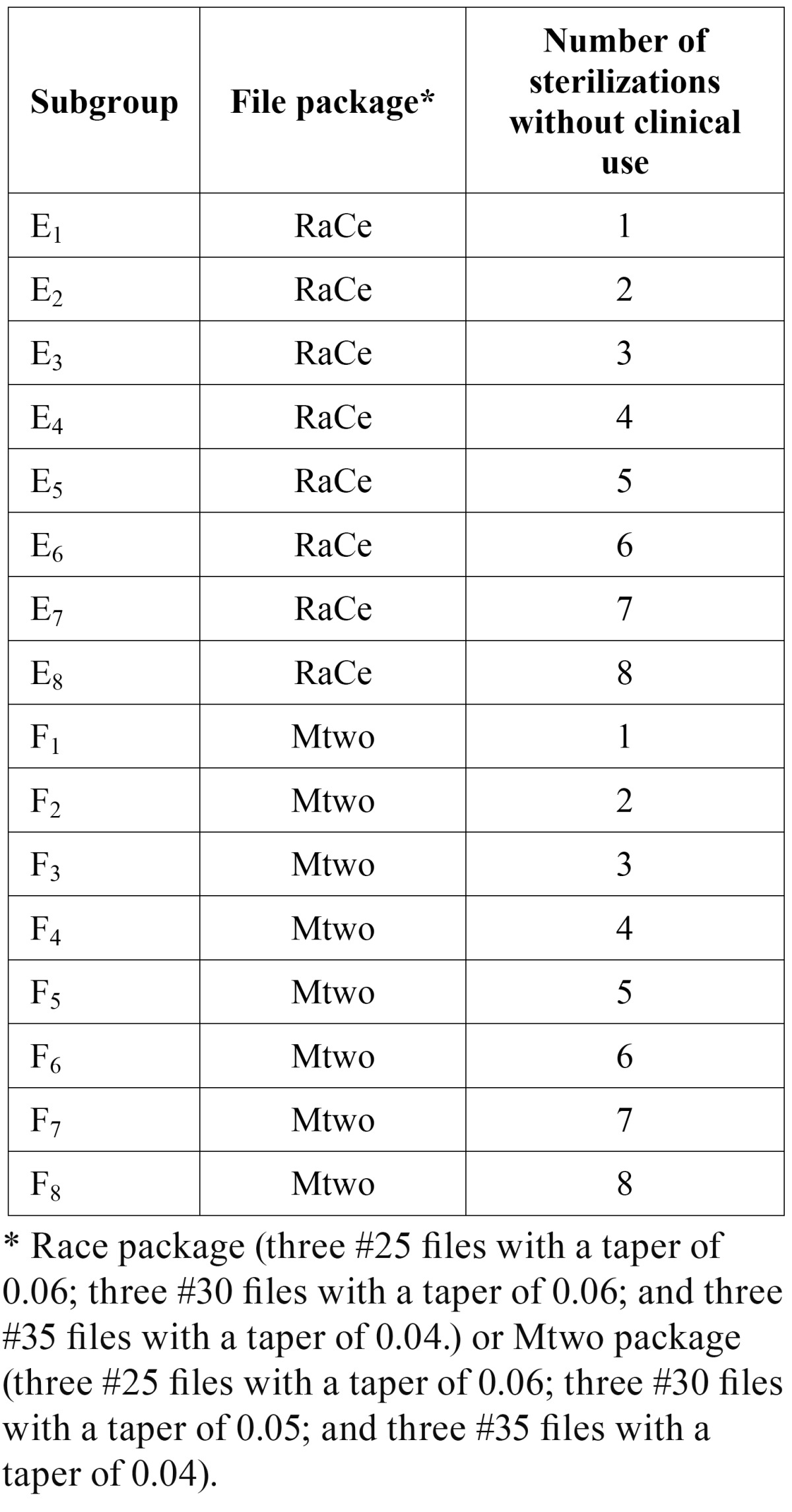
Autoclave subgroups and number of sterilization procedures used (n=144).

3. Control group: Two new RaCe and Mtwo file packages consisting of 9 files, similar to the ones used in the working and autoclave groups, were used as controls (n=18). These files were dispensed from their original packages and used in the corrosion analysis without being used, disinfected and autoclaved.

Finally, all the files in all the groups were subjected to corrosion analysis (electrochemical measurement).

-Electrochemical measurement

Electrochemical Impedance Spectroscopy (EIS) is one of the most frequently used techniques for providing information about corrosion of metals. The complex impedance generated by EIS is often analyzed by adaptation to the response of an equivalent electrical circuit composed of passive elements such as resistors, capacitors and inductors, thereby determining various characteristic properties of the metals and surface coatings. Low-frequency impedance is commonly used to predict corrosion resistance.

**Figure 1:**
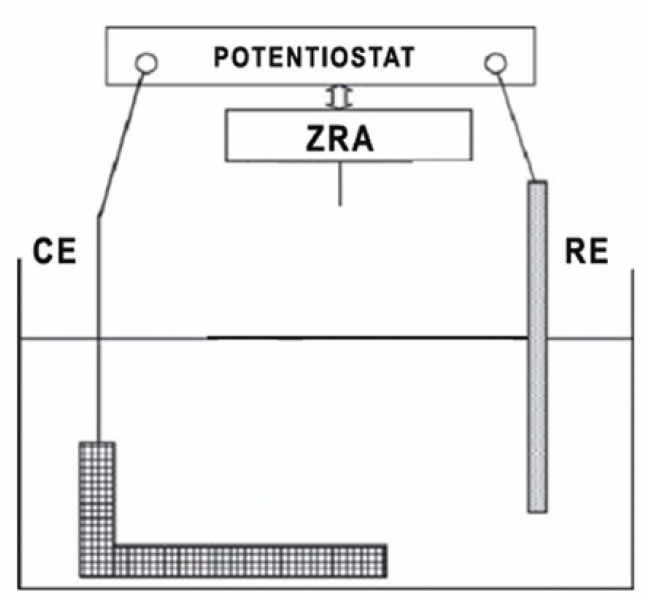
The working electrodes were the rotary instruments individually coupled through the zero resistance ammeter (ZRA). The potentiostat derives the current through platinum gauze acting as counter electrode (CE), and measures the potential of the galvanic system with respect to the reference electrode (RE).

Electrochemical measurements were performed using a potentiostat (Applied Corrosion Monitoring, Cark-in-Cartmel, UK) equipped with a ZRA (zero resistance ammeter) (Fig. 1) for galvanic current measurements. A large surface platinum gauze was employed as a counter electrode. The reference electrode employed was an Ag/AgCl electrode with an electrochemical potential of 16 mV with respect to the saturated calomel electrode (SCE). The working electrodes were the rotary instruments individually coupled through the potentiostat’s ZRA. All the electrochemical tests were performed at room temperature (22 ± 2ºC). The files were embedded along their longitudinal axis in an epoxy resin and their corrosion behavior was determined electrochemically by cyclic potentiodynamic polarization which is capable of measuring localized corrosion susceptibility of nickel-titanium alloys. Before testing, the specimens were cleaned in an ultrasonic bath with distilled water and were left to dry. Then, they were placed in the polarization cell for 24 h before initiating polarization. Polarization curves were obtained by using a potentiostat.

The experiment was designed to determine changes in corrosion resistance of rotary files as a function of these factors: file type (Mtwo vs. RaCe) and irrigation solutions (NaOCl vs. NaCl) and multiple cycles of sterilization and root canal preparations. Normal distribution of data was tested by Shapiro-Wilk test and assumptions derived from the tests were examined by non-parametric tests. Mann-Whitney U test was used for independent groups and Wilcoxon’s test was used for dependent groups. Statistical significance was set at p< 0.05.

## Results

-Part I: Working, autoclave and control groups

Statistical analysis indicated that there were significant differences in corrosion resistance of files associated with working and autoclave groups between RaCe and Mtwo file types (p<0.001). Statistical analysis also indicated that there were significant differences in corrosion resistance of files associated with working and control groups between the two file types (p<0.001). However, there were no significant differences in corrosion resistance of RaCe and Mtwo files between the autoclave and control groups (p=0.344).

In the autoclave group statistical analysis indicated that, in RaCe type only, corrosion resistance difference was significant between the autoclave and working groups (p<0.001), and the working and control groups (p<0.001); however, there was no difference in corrosion resistance between the autoclave and control groups (p=0.259). In Mtwo type only, the differences between the groups were similar to those in RaCe type, but it was observed, contrary to what was expected, that corrosion resistance of Mtwo rotary files between the autoclave and control groups was significant (p=0.009). Corrosion resistance for Mtwo files was significantly higher than that in RaCe type and in the working groups corrosion rate was significantly higher than that in the autoclave and control groups. Corrosion rate was under the influence of multiple cycles of preparation and sterilization (Figs. 2A,2B,2C).

**Figure 2A:**
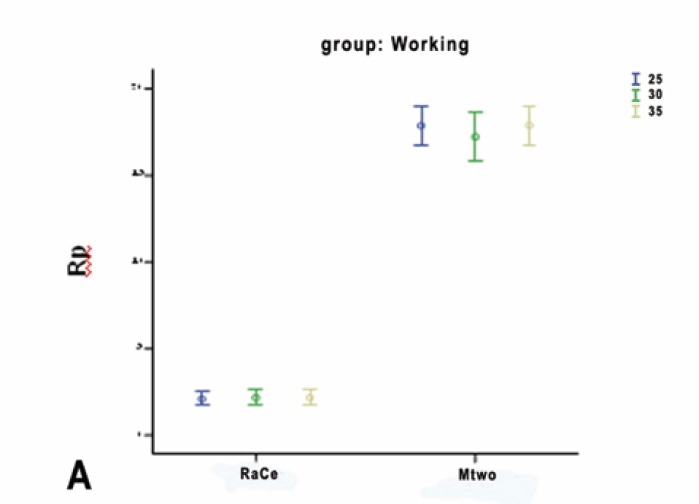
Error bars of corrosion resistance, which illustrate the mean standard deviation, minimum, and maximum amount of corrosion resistance of files (sizes: 25, 30, 35) in A) working.

**Figure 2B:**
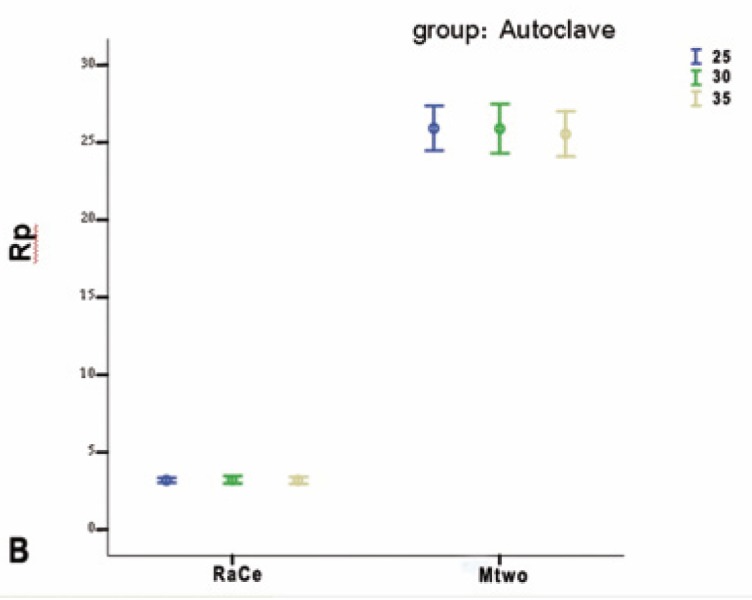
Autoclave.

**Figure 2C:**
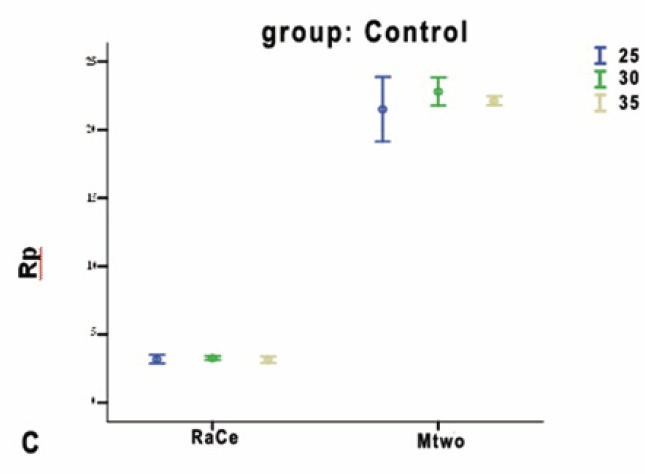
Control groups.

-Part II: Irrigation solutions in the working groups

Statistical analysis indicated that there were significant differences in corrosion resistance of files associated with NaOCl and NaCl in the working group in RaCe file type (p<0.001). In Mtwo file type there were no significant differences in corrosion resistance of files associated with NaOCl and normal saline in the working group (p=0.889). Changes in corrosion resistance for RaCe files was higher in NaOCl than those in normal saline, but corrosion for Mtwo files was not under the influence of irrigation solutions.

-Part III: Effect of multiple cycles of preparation and sterilization procedures

**Figure 3 A,B:**
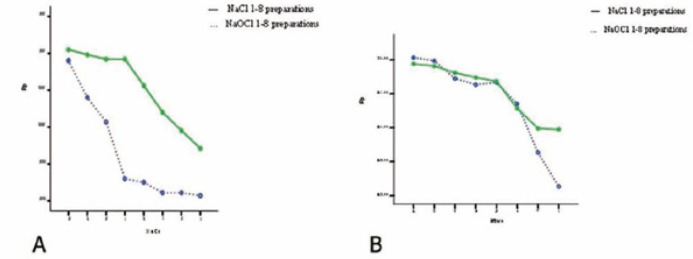
Trend of corrosion resistance changes for RaCe A) and Mtwo B) files (1-8 uses).

Fig. 3 A,B summarizes the initial corrosion resistance change of RaCe and Mtwo files according to the number of preparations with a series of files in NaOCl and NaCl irrigation solutions.

The results showed that the number of sterilization procedures negatively influences corrosion resistance of RaCe files; in contrast, in Mtwo files this relationship was positive and sterilization cycles increased corrosion resistance of Mtwo files.

There were no statistically significant differences between different sizes of files in Mtwo and RaCe types in different irrigation solutions (p=0.10).

Comparison of corrosion resistance based on the number of uses in sodium hypochlorite and normal saline solutions in RaCe files showed that after preparation of 4 and 5 canals, respectively, with one instrument corrosion resistance significantly decreases. The same comparison in Mtwo files showed that in using both solutions, after 4-8 canal preparations, a gradual decrease in corrosion resistance was observed in every series of files.

Comparison of corrosion resistance after 1-8 sterilization procedures in RaCe files showed that after 4-8 sterilization procedures, corrosion resistance gradually decreases; in contrast, the same comparison in Mtwo files showed that after 4 sterilization procedures the files exhibit an increase in corrosion resistance.

There were no statistically significant differences between different sizes of#25, #30, and #30 in Mtwo and RaCe file types in different irrigation solutions (p=0.10). Size was not an important variable in corrosion resistance changes of files in this study.

Comparison of corrosion resistance of RaCe and Mtwo files in autoclave groups based on file sizes did not reveal any statistically significant differences between them (p=0.12).

## Discussion

Corrosion resistance of root canal instruments influences their clinical behavior. It is likely that pitting or crevice corrosion occurs initially and then increases the odds of fatigue failure, thereby changing the fracture mechanism from conventional fatigue failure to corrosion failure. The present study evaluated the effect of irrigation solutions, multiple cycles of sterilization procedures, and root canal preparations on nickel-titanium rotary files from two different manufacturers, and determined whether resistance to corrosion was under the influence of these factors. The results of this study showed significant effect of NaOCl on RaCe rotary files. However, NaOCl did not significantly influence corrosion resistance of Mtwo files. This finding is consistent with the results of a study carried out by Topuz et al. (13), who reported visible corrosion on RaCe files in NaOCl in a time-dependent pattern; however, Haïkel et al. (15) found no significant effect on NiTi files after immersion in 2.5% NaOCl for 12 to 48 h. In this study 2.5% NaOCl at 50°C was used because it has been demonstrated that a higher temperature increases its potential to dissolve organic substrates (16,17). Furthermore, in the present study, chelation with RC-prep was used for all the files. Darabara et al. (18) showed that pitting or crevice corrosion of endodontic files are not likely to occur in chelating solutions such as R-EDTA.

In another part of this experiment, the results demonstrated that the number of sterilization procedures negatively affects corrosion resistance of RaCe files, which is consistent with the results of other studies (19-23). In contrast, it is surprising that in Mtwo files this relationship is positive and sterilization cycles increases corrosion resistance of Mtwo files, as found in the present study, where Mtwo files demonstrated a significantly higher resistance to corrosion compared to RaCe files after canal preparation. All these observations might be attributed to differences in the chemical composition of metals used for manufacturing the RaCe and Mtwo files, which are not usually revealed by manufacturers. Stokes et al. (6) speculated that manufacturing factors affect corrosion of NiTi endodontic instruments.

In addition, the presence of surface irregularities on NiTi files (24) may compromise their cutting efficiency (25) and probably make them more susceptible to corrosion and fracture (26). The s-shaped cross-sectional design of Mtwo files results in very aggressive cutting edges and a positive rake angle, which is known to require less energy to cut dentin than blades with a neutral or negative rake angle (27), leading to less crack and surface irregularities on the file, which increases corrosion resistance. Furthermore, one contributing mechanism for increased corrosion resistance after sterilizations in Mtwo instruments may be the alteration of the phase transformation for the NiTi alloy, including the decrease in the martensitic temperature to subambient levels to yield a completely austenitic structure at room temperature. However, this is controversial, and further studies are required to investigate alloy behaviour under clinical conditions.

In conclusion, within the limitations of this study, corrosion resistance was significantly higher in Mtwo files compared to RaCe files in sizes #25, #30, and #35 and these properties might affect clinical efficacy of these instruments, necessitating further investigations. One should keep in mind that this study only studied two of the several file systems available and only 3 sizes of files were used. Therefore, to draw a more definitive conclusion on the subject, further research is warranted with multiple brands of files in multiple sizes and in different methods to detect corrosion.
